# Case of Perforation of the Bladder, and Successive Formation of Calculi

**Published:** 1827-10

**Authors:** P. L. Serph

**Affiliations:** Surgeon, Welch Pool.


### PERFORATION OF THE BLADDER.

*Case of Perforation of the Bladder, and successive Formation of Calculi.* By P. L. Serph, Surgeon, Welch Pool. (Communicated by Dr. James Johnson.)

Mrs. A., aged thirty-five years, a married woman, having borne a child in 1818, and miscarried in 1821, enjoying general good health till the month of January, 1823, when she was seized with severe pain in the loins, which at the time was supposed to be lumbago, and lasted about fourteen days. In May following, Mrs. A. was attacked with acute pain in the two hip-joints at the same time, the severity of which confined her one week in bed, and caused a lameness, which lasted about three weeks; but the pain never went away entirely till the autumn of 1826. In August of the same year (1823) the patient discovered for the first time a small tumor, loose to the touch, but deeply seated in the thickness of the labia pudendi of the left side, which grew till the following month of August 1824, without causing any pain, except during a few days previously to its bursting, which it did about that period, and discharged strumous matter. From this time it never healed, and a constant oozing was kept up from the part.

In March, 1826, Mrs. A. felt for the first time symptoms of irritation in the bladder, and tried various means to obtain relief, but in vain. In November I was called in, and, from the symptoms, suspected the presence of a calculus in the bladder, which the introduction of a female catheter on the 18th of November proved to be correct. Of course, the first thing to be done was to remove it, and I fixed upon the plan lately adopted by some eminent surgeons of dilating the urethra. I procured Mr. Weiss's dilator, and on the 29th attempted the operation for the first time, and in about twenty minutes dilated the urethra sufficiently to admit the introduction of a finger into the bladder, that I might ascertain with more precision (which I easily did) the situation, &c. of the calculus. I then introduced the forceps usually employed in lithotomy, but could not seize the stone with it, and after three or four fruitless attempts I put it aside; and, having again with my finger brought the calculus forward, I seized and removed it very easily with the common dressing forceps, the branches of which I had previously slightly curved on each other. The stone was of the mulberry kind, about the size and shape of a peach-stone. During the dilatation, some small vessel gave way on the surface of the urethra, and a few ounces of venous blood were lost. The patient was put into a hip-bath, and an opiate administered. No accident occurred; and the bladder never for an instant lost its retentive and expulsive powers, but it remained very irritable, and Mrs. A. complained of a constant and fixed pain in the left side of the lower part of the abdomen; and a discharge of matter, having a muco-purulent appearance, took place when the urine was evacuated, which was more abundant in the morning, and then of a very fetid smell.

From the above-mentioned symptoms, and others incident on ulceration of the bladder, I directed my plans accordingly, and made use of the various remedial means usually employed in ulceration of that viscus, but in vain.

After improving during a short time, the symptoms again became more distressing, and the patient positively asserted that there was another stone in her bladder, but it could not be detected by the sound. On the 6th of May, 1827, the sensations became so similar to those she had felt before the removal of the first stone, that she herself introduced the catheter, and the percussion of the instrument against a calculus proved her judgment to have been correct. On the 7th, I proceeded to another operation, which was very tedious, in consequence of the stone being so extremely soft that it crumbled like half-dried mortar between the blades of the forceps; and, having removed every fragment the instrument could detect, I washed away the remaining particles by means of the enema syringe, invented by Mr. Read. I again explored the bladder with my fore-finger, and, not being able to detect any other calculus, I flattered myself and my patient with the hope of her obtaining by this last operation a radical cure.

All went on promisingly for a short time, when the pain within the left side of the bladder again returned, and all the former symptoms with it, which became more and more distressing. Early in July the unfortunate patient asserted again that there was another calculus, and submitted to a third operation, which was performed on the 4th instant. Having sufficiently dilated the urethra, I introduced the fore-finger of the right hand into the bladder, repeatedly explored its fundus and side, endeavoured to discover if any cyst existed that might contain a calculus, and, never suspecting that a stone could adhere at the anterior and superior part of that viscus, I was on the point of giving up the search, when for the first time a suspicion of the connexion between the fistulous opening on the labia and the os pubis suggested itself, and, turning the palm of my hand upwards, that I might bring the finger towards the posterior surface of the pubis, I immediately detected a rough substance, part of which crumbled under my touch. I endeavoured to loosen the remainder, which was so rough and so hard that I was obliged to desist, from the pain it gave me. I then introduced a lever (something similar to those employed in raising depressed portions of the cranium, after using the trephine,) previously curved to an angle of about 45°, twice fixed it on the hard substance, and applied to it a force much more than sufficient to break it in pieces had it been of a consistency even much harder than urinary calculi generally are, but all in vain. In the last attempt, a piece of irregular shape broke off, which was of a dark colour, and extremely hard. Between the two attempts with the lever and after the last, I introduced my finger, and, in endeavouring to shake the substance, it felt of that kind of looseness which we sometimes feel in shaking between the finger and thumb some of the molares, which, however, require a great power to extract them.

It appears evident, first, that, so long as 1823, the disease began to develop itself in the os pubis of the left side, and that that bone became carious;—secondly, that an abscess formed, which interested externally the labia of that side, and internally found its way through the coats of the bladder within that viscus;—thirdly, that an osseous excrescence, arising from the pubis, penetrated through that opening, and became a nucleus, on the surface of which the calcareous part of the urine was in part deposited, and that concretions were made in the same manner as stalactites are formed;—fourthly, that, when the concretion had acquired a certain size, it detached itself, and became a common urinary calculus, loose in the bladder;—fifthly, that, had I succeeded in breaking off the bony process, accidents might have arisen from the contact of the urine with a sound part of the bone.

Now, sir, allow me to ask you and the profession, through your valuable Journal, ought the case to be abandoned to nature, except removing out of the bladder the calculi which may be there deposited? what would be the most likely plan to obtain a cure? and what prognosis ought to be drawn from our knowledge of the case? There is not the least doubt that the original cause of the disease was scrofula. I have since the last operation desisted from all remedial means except the Tincture of Iodine; and I have the pleasure to say, that she now feels better and much more comfortable than she has done during the last twelve months. Her general health is certainly much improved.

I cannot close this statement without paying a just tribute of praise to the ingenuity of Mr. Weiss, whose talent in executing the plans formed by surgeons cannot be too much commended. I am convinced the use of his dilator of the female urethra will soon supersede every method formerly employed in removing urinary calculi in all cases where females are afflicted with that disease.

*Welch Pool; July 31st, 1827.*

